# Oncogene Downregulation by Mahanine Suppresses Drug-Sensitive and Drug-Resistant Lung Cancer and Inhibits Orthotopic Tumor Progression

**DOI:** 10.3390/cancers16213572

**Published:** 2024-10-23

**Authors:** Raghuram Kandimalla, Disha N. Moholkar, Suman Kumar Samanta, Neha Tyagi, Farrukh Aqil, Ramesh Gupta

**Affiliations:** 1 Brown Cancer Center, University of Louisville, Louisville, KY 40202, USA; raghuram.kandimalla@louisville.edu (R.K.); dishanagesh.moholkar@louisville.edu (D.N.M.); farrukh.aqil@louisville.edu (F.A.); 2 Department of Pharmacology & Toxicology, University of Louisville, Louisville, KY 40202, USA; 3 Faculty of Science, Assam Down Town University, Guwahati 781026, India; suman.s@adtu.in; 4 Department of Medicine, University of Louisville, Louisville, KY 40202, USA

**Keywords:** mahanine, NSCLC, polypharmacology, oncogenes, apoptosis, cell cycle arrest

## Abstract

**Background/Objectives**: Lung cancer is one of the deadliest cancers, and drug resistance complicates its treatment. Mahanine (MH), an alkaloid from *Murraya koenigii* has been known for its anti-cancer properties. However, its effectiveness and mechanisms in treating non-small cell lung cancer (NSCLC) remain largely unexplored. The present study aimed to investigate MH’s effect on drug-sensitive and drug-resistant NSCLC and its potential mechanism of action. **Methods**: We isolated MH from *M. koenigii* leaves and the purity (99%) was confirmed by HPLC, LC-MS and NMR. The antiproliferative activity of MH was determined using MTT and colony formation assays against drug-sensitive (A549 and H1299) and Taxol-resistant lung cancer cells (A549-TR). Western blot analysis was performed to determine MH’s effects on various molecular targets. Anti-tumor activity of MH was determined against lung tumors developed in female NOD Scid mice injected with A549-Fluc bioluminescent cells (1.5 × 10^6^) intrathoracically. **Results**: MH dose-dependently reduced the proliferation of all lung cancer cells (A549, H1299 and A549-TR), with IC50 values of 7.5, 5, and 10 µM, respectively. Mechanistically, MH arrested cell growth in the G0/G1 and G2/M phases of the cell cycle by inhibiting cyclin-dependent kinase 4/6 (CDK4/6) and cell division control 2 (CDC2) and induced apoptosis through the downregulation of B-cell leukemia/lymphoma 2 (BCL2) and B-cell lymphoma-extra large (BCL-XL). The apoptotic induction capacity of MH can also be attributed to its ability to inhibit pro-oncogenic markers, including mesenchymal–epithelial transition factor receptor (MET), phosphorylated protein kinase B (p-AKT), phosphorylated mammalian target of rapamycin (p-mTOR), survivin, rat sarcoma viral oncogene (RAS), myelocytomatosis oncogene (cMYC), and nuclear factor kappa-light-chain-enhancer of activated B cells (NF-κB) levels. In vivo, MH (25 mg/kg b. wt.) significantly (*p* < 0.001) inhibited the growth of A549 lung cancer orthotopic xenografts in NOD Scid mice by 70%. **Conclusions**: Our study provides new mechanistic insights into MH’s therapeutic potential against NSCLC.

## Simple Summary

Lung cancer is the deadliest of all cancer types and often develops resistance to chemotherapy, necessitating the search for new therapeutic options. In this context, the current study aimed to demonstrate the application of mahanine (MH), a plant-derived phytochemical from the curry leaf, as a potential treatment for drug-sensitive and drug-resistant lung cancer, while also proposing a potential mechanism of action. The results of this study establish MH as a potential treatment option for both drug-sensitive and -resistant lung cancer. Furthermore, this study may serve as a foundation for research into specific oncogene regulation and the potential use of MH as an adjuvant therapy alongside other chemotherapeutics for lung cancer patients.

## 1. Introduction

Lung cancer remains the deadliest of all cancers, contributing significantly to cancer-related deaths worldwide. In 2023, the United States alone witnessed 238,240 new cases of lung cancer, resulting in 127,070 deaths [1]. Non-small cell lung cancer (NSCLC) accounts for about 85% of all lung cancer cases. While treatments for lung cancer, including surgery, chemotherapy, radiation therapy, and immune checkpoint inhibitors, have advanced significantly in recent years, the five-year survival rate remains low at 23%, positioning lung cancer as the leading cause of cancer-related death [1]. Moreover, drug resistance poses a significant challenge in treating NSCLC patients [2,3]. Patients undergoing chemotherapy gradually acquire genetic alterations with each clonal proliferation of tumor cells. The activation of proto-oncogenes and inactivation of tumor-suppressor genes are attributed to these mutations. The treatment of the tumors becomes more complicated when the tumors become drug-resistant. For instance, patients with NSCLC frequently receive paclitaxel; however, prolonged therapy with this drug often leads to resistance and subsequent therapeutic failure, worsening patient prognosis [2,3]. Therefore, new therapeutic approaches are urgently needed to treat both drug-sensitive and drug-resistant NSCLC.

Conversely, the transition of normal cells into malignant counterparts is primarily driven by mutations in proto-oncogenes such as mesenchymal-epithelial transition factor receptor (MET), kirsten rat sarcoma viral oncogene homolog (KRAS), epidermal growth factor receptor (EGFR), and v-raf murine sarcoma viral oncogene homolog B, among others [4]. These genetic alterations predominantly promote tumorigenic characteristics such as angiogenesis, invasion, metastasis, and proliferation in cancer cells. Furthermore, the oncogenic transcription factor nuclear factor kappa-light-chain-enhancer of activated B cells (NF-κB) has emerged as a pivotal contributor to NSCLC development. Studies indicate significant histological heterogeneity in lung tumor samples from patients; however, the majority exhibit elevated levels of NF-κB, highlighting its significance as a therapeutic target for NSCLC [5]. Recognizing these molecular intricacies, the United States Food and Drug Administration has approved several drugs designed to target at least seven distinct lung cancer mutations [6], alongside NF-κB-targeting therapies [5]. Despite these advancements, lung cancer remains a complex disease with multiple contributing factors and genetic abnormalities that influence prognosis. Existing therapies typically address one or two of these abnormalities, leaving scope for innovative pharmacological approaches capable of targeting multiple aberrant pathways simultaneously.

Phytochemicals found in various foods and natural products offer promising new avenues for novel drug discovery. Small molecules derived from medicinal plants have long been utilized in cancer treatment by targeting multiple pathways. Among these plant secondary metabolites, glycosides, phenolics, and alkaloids have garnered attention for their medicinal properties. Mahanine (MH), a carbazole alkaloid isolated from the leaves of *Murraya koenigii*, is recognized for its anticancer potential [7]. Previous research indicates its efficacy in preventing various cancer types, including lymphoma, colorectal, breast, and prostate cancers, through mechanisms such as apoptosis and cell cycle arrest.

We previously reported the anticancer potential of MH against different breast cancer subtypes [8], demonstrating its ability to inhibit tumor growth by modulating cell cycle genes, such as cyclin-dependent kinase 4 (CDK4) and cyclin-dependent kinase 6 (CDK6), and arresting the G_0_/G_1_ phase in breast cancer cells. Despite some studies, the role of MH in controlling the growth of NSCLC remains largely unexplored. Building on our prior investigations into MH’s anticancer effects, the primary objective of this study was to assess its potential against both drug-sensitive and drug-resistant NSCLC using in vitro and in vivo models as well as to elucidate its mechanisms.

## 2. Materials and Methods

### 2.1. Chemicals and Reagents

Cell culture media, fetal bovine serum (FBS), trypsin–EDTA solution (0.25%), and antibiotic solution (Pen/Strep) were purchased from Thermo Fisher Scientific (Waltham, MA, USA). Crystal violet and 3-(4,5-dimethylthiazol-2-yl)-2,5-diphenyltetrazolium bromide (MTT) were obtained from Sigma-Aldrich (St. Louis, MO, USA). IVIS brite D-luciferin potassium salt bioluminescent substrate (Luciferin) was purchased from Perkin Elmer (Waltham, MA, USA). Primary and secondary antibodies, like rat sarcoma viral oncogene (RAS), phosphorylated mammalian target of rapamycin (p-mTOR), myelocytomatosis oncogene (cMyc), phosphorylated protein kinase B (p-AKT), MET, survivin, β-Actin, HRP-linked anti-mouse and anti-rabbit, CDK4, CDK6, cell division control 2 (CDC2), and NF-κB used for western blot analyses were purchased from Cell Signaling Technology (Danvers, MA, USA) and Santa Cruz Biotechnologies (Dallas, TX, USA). Solvents used for the extraction of MH and all other reagents were of an analytical grade.

### 2.2. MH Isolation, Purification, and Characterization

**Figure 1:**
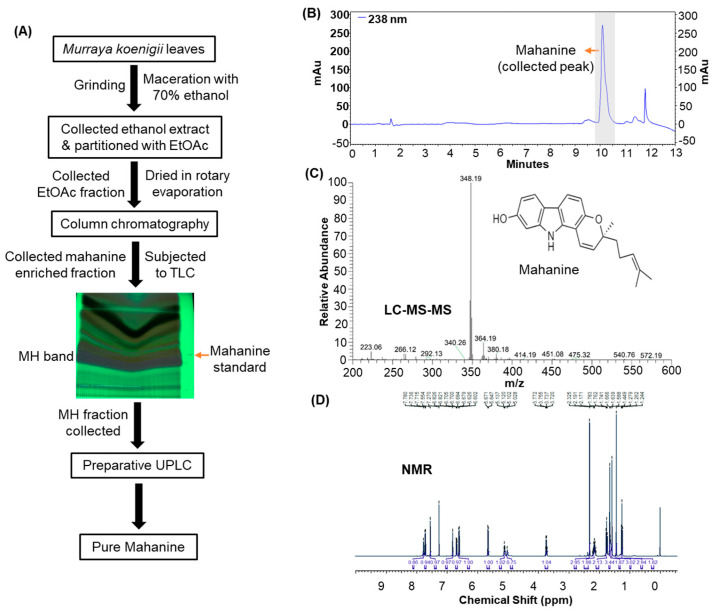
Isolation and purification of mahanine (MH) from *Murraya koenigii* leaves. (**A**) The scheme depicts the isolation of MH from leaves via maceration with hydroalcoholic extraction, followed by partitioning and phase separation with ethyl acetate. Further entrenchment of MH fractions was performed using column and thin-layer chromatography, followed by preparative UPLC. (**B**) UPLC chromatogram shows the MH peak isolated in larger quantities. (**C**) Mass spectroscopy chromatogram of MH in positive-ion mode showed *m*/*z* of 348.19. The inset represents the chemical structure of MH. (**D**) ^1^H NMR spectra of MH isolated for this study were recorded at 600 MHz.

*Murraya koenigii* leaves were collected from southern India (15.828521° N, 80.188869° E) in September 2020. The leaves were cleaned and shade dried. The leaves were finely ground into powder and extracted with 70% ethanol through maceration for 72 h. The extract was collected by straining through muslin cloth, followed by filtration using Whatman No. 2 filter paper. The extract was concentrated using a rotary evaporator and then thoroughly mixed and partitioned with ethyl acetate. The phases were separated using a separating funnel, and the ethyl acetate fraction was collected, concentrated using a rotary evaporator, mixed with silica (mesh size 100–200), and subjected to column chromatography. Petroleum ether and chloroform mixed in the ratio of increasing polarity were used to elute different fractions, as described in [9]. MH in the different eluates was confirmed by analytical thin-layer chromatography (TLC) and ultra-performance liquid chromatography (UPLC) analysis (Figure 1A). To further purify MH, preparative silica-gel TLC, followed by preparative UPLC, was performed. Mass spectrometry and ^1^H Nuclear magnetic resonance (NMR) analyses were conducted to confirm the identity of MH.

#### 2.2.1. Preparative TLC

MH-enriched fractions from column chromatography were pooled and dried using a rotary evaporator. The dried MH-enriched fraction was dissolved in ethanol and loaded onto preparative TLC plates (Sigma; cat No.: 60778). The plates were developed using a mixture of chloroform and methanol (95:5) containing 0.1% formic acid (*v*/*v*). An MH reference was included to compare the retention time (RT) of the sample. After development, the plates were dried, and the MH band on the silica plate was scraped off and extracted with ethanol. Following filtration through the Whatman No. 2 filter paper, the MH-enriched fraction was further purified by preparative UPLC.

#### 2.2.2. Preparative HPLC

Preparative HPLC analysis was carried out using the method of Kandimalla et al. [10] with modification on a Shimadzu preparative liquid chromatography system (Kyoto, Japan). The MH-enriched fraction (20 mg in 200 μL volume) was injected into an Apollo C18 preparative column (particle size, 5 µm; length, 250 mm; diameter, 10 mm) using methanol and water (9:1 *v*/*v*) as the mobile phase at 10 mL/min flow rate. The MH peak (16–19 min) was collected using an automated sample collector, dried, and stored at −80 °C until use.

#### 2.2.3. Mass Spectroscopy Analysis

To assess the purity of isolated MH, liquid chromatography–mass spectrometry (LC-MS) analysis was performed using ExACTIVE PLUS Ultimate 3000 ultra-high pressure liquid chromatography coupled with an MS (Thermo Fisher Scientific, Waltham, MA, USA). The chromatographic analysis was carried out on a Hypersil Gold C-18 column (particle size, 1.9 μm; length, 150 mm; diameter, 2.1 mm). A 10 μL sample (5 mg/mL) was injected, and the mass spectrum was recorded on the MS with electrospray ionization in positive mode.

#### 2.2.4. NMR Analysis

HPLC-purified MH was dissolved in chloroform, and proton NMR spectra were recorded using a 400 MHz NMR instrument (Varian, Model: Mercury plus) at the Indian Institute of Technology Guwahati, India. The acquired data were compared with a reference compound and previously reported results [9] to ensure consistency and molecular integrity and detect any potential contamination.

### 2.3. Cell Culture and Maintenance

Human lung cancer cell lines A549 and H1299 were received from Dr. Gairola (University of Kentucky, Lexington, KY, USA) and Dr. Paula Bates (University of Louisville, Louisville, KY, USA), respectively. Taxol-resistant lung cancer cells (A549TR) were generously provided by Dr. Bruce Zetter of Children’s Hospital Boston, Harvard Medical School (Boston, MA, USA). Bioware^®^ Brite Cell Line A549 Red-FLuc (A549-Luc) was purchased from PerkinElmer, Waltham, USA. A549 and A549-TR cells were cultured in RPMI (Gibco, Waltham, MA, USA), and H1299 was cultured in DMEM (Gibco, Waltham, MA, USA) supplemented with 10% FBS and 1% antibiotic (penicillin/streptomycin) solution. For A549-Luc cells, RPMI supplemented with 10% FBS and puromycin at 2 µg/mL was used.

### 2.4. Cell Viability Assay

#### 2.4.1. MTT Assay

The effect of MH on the proliferation of lung cancer cells was determined by an MTT assay. Briefly, lung cancer cells (A549, A549-TR, and H1299) were seeded in 96-well plates at a density of 3000 cells/well and allowed to attach for 24 h at 37 °C in 5% CO_2_. The next day, the media was replaced with fresh media containing varying concentrations of MH. After incubation for 72 h, the media was replaced with fresh media containing 10% MTT (final concentration of 0.5 mg/mL) and incubated for 3 h. The media was aspirated, and 200 µL dimethyl sulfoxide was added to each well to dissolve the formazan crystals, and the absorbance was measured at 570 nm [11].

#### 2.4.2. Luminescence Assay

The ability of MH to inhibit the growth of lung cancer luciferase-expressing A549-Luc cells was determined by a luminescence assay. Briefly, A549-LUC cells (3 × 10^3^ cells/well) were seeded in 96-well white plates. After 24 h incubation at 37 °C in 5% CO_2_, the media was replaced with drug-containing media, and incubation was continued for 72 h. After treatment, the media was changed with luciferin (150 μg/mL) containing media for 10 min. Luminescence intensity was measured by a SpectraMax spectrophotometer [11].

#### 2.4.3. Colony Formation Assay

The ability of MH to inhibit colony formation by lung cancer cells (A549, A549-TR, and H1299) was assessed by a clonogenic assay. Lung cancer cells (500 cells per well) were seeded in 6-well plates. After 24 h, the culture media was changed with fresh media containing various concentrations (2.5 to 20 μM) of MH for 24 h. The drug-containing media was replaced with fresh media 24 h after treatment, and the plates were incubated for the next 10 day at 37 °C in a CO_2_ incubator. During incubation, media was replaced every other day. On day 11, the culture media was removed, and cells were fixed in 100% methanol for 15 min. Cells were then stained with 0.05% crystal violet (dissolved in methanol) by incubating for 60 min at room temperature. The cells were repeatedly rinsed with distilled water until the background was clean. The number of colonies in different treatments was counted manually [11].

### 2.5. Western Blotting Analysis

The effect of MH on lung cancer oncogenes, cell cycle, and apoptotic markers was assessed through Western blot analysis. Briefly, A549, A549-TR, and H1299 cells (150 K cells/well) were plated in a 12-well plate and allowed to attach overnight. The next day, cells were treated with various concentrations (2.5–20 μM) of MH for 48 h. Cell lysates were prepared using RIPA lysis buffer (Santa Cruz Biotechnologies, Dallas, TX, USA) supplemented with protease and phosphatase inhibitors. Western blot analysis was performed, as described previously [12], and the blots were probed for CDK4, CDC2, CDK6 (Cell cycle analysis), BCL-2, BCL-XL (apoptosis analysis) and MET, p-AKT, p-mTOR, survivin, RAS, and cMyc (oncogenic markers). β-Actin was used as a loading control. All the primary and secondary antibodies tested were used at 1:1000 dilution except for the loading control β-Actin (1:10,000).

### 2.6. Preparation of Nuclear Extracts for NF-κB Assessment

NF-κB plays a role in many biological processes, including inflammation, cell growth, and survival. For the analysis of NF-κB, nuclear extracts from A549, A549TR, and H1299 cells treated with a vehicle or MH for 48 h were prepared as described previously [13]. The protein concentration was determined by BCA analysis following the manufacturer’s instructions. The expression of NF-κB was assessed by Western blotting. Lamin-B1 was used as a loading control.

### 2.7. Effect of MH on Orthotropic Lung Tumors

Female NOD Scid mice (5–6 weeks old) were obtained from Charles River laboratories. The animal study was carried out in strict accordance with the protocol approved by the Institutional Animal Care and Use Committee (IACUC) of the University of Louisville (approval number: 23270). The animals were acclimatized on normal chow for 7 d. The chow diet was then substituted with a purified AIN93M diet for the study duration.

Animals were inoculated with Bioware^®^ Brite A549 Red Fluc cells (1.5 × 10^6^ cells) in 50 µL of Matrigel (BD Bioscience, Bedford, MA, USA) mixed with serum-free media (1:1; *v*/*v*) via intrathoracic injection using 30-gauge needles, as described previously [11]. Animals were provided an AIN-93M purified diet and water ad libitum. Tumor growth was assessed by measuring luciferase intensity 15 min post intraperitoneal injection of the luciferin (120 mg/kg; 100 µL). After 10 days, when the luminescence intensity reached approximately 1 × 10^6^ photons, animals were divided into two groups (*n* = 8–10) and treated with a vehicle or MH (25 mg/kg) intraperitoneally three times a week. The luciferase signals were detected using an in vivo imaging system manufactured by Biospace Lab. When the average luminescence intensity in the vehicle treatment reached approximately 2 × 10^8^ photons, all the animals were euthanized. Tumors, lungs, and livers were weighed, and blood serum was collected. All the tissues and serum were stored at −80 °C until analyzed.

## 3. Results

### 3.1. MH Isolation and Characterization

The ethanolic extract from *M. koenigii* leaves was dried and lyophilized, which yielded a total of 5.2% dried extract. The ethanolic extract showed several peaks, with the MH peak corresponding to 6.4% of the total extract. Further fractionation of the ethanol extract with ethyl acetate resulted in a 14.2% ethyl acetate fraction, with the purity of MH improving to 32%. To further purify MH, the ethyl acetate fraction was applied to silica gel column chromatography and preparative TLC and was finally purified by preparative UPLC, resulting in MH with >99% purity. A total of 304 μg (30.4%) of purified MH was recovered per mg of the ethyl acetate fraction. Figure 1B shows the UPLC chromatogram of purified MH. The mass of the isolated MH was 347.18, as measured by mass spectroscopy in positive mode, confirming that the isolated compound is MH (Figure 1C). Furthermore, ^1^H NMR analysis reveals that the isolated substance contained 25 H atoms, confirming that it is, indeed, MH (Figure 1D).

### 3.2. MH Inhibits Lung Cancer Cell Proliferation

#### 3.2.1. Effect on Cell Viability

**Figure 2:**
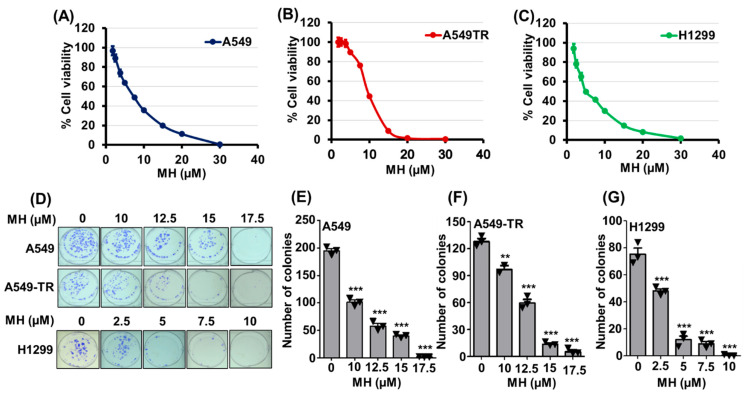
Effect of mahanine (MH) on lung cancer cell proliferation. Cell viability of A549 (**A**), A549-TR (**B**), and H1299 (**C**) was determined by MTT assay after treatment with MH for 72 h. Data represent mean ± S.D. of 3 independent experiments. MH dose-dependently inhibited the colony-forming ability of NSCLC cells. (**D**) Images depict the colony-forming assay of drug-sensitive (A549 and H1299) and drug-resistant (A549-TR) NSCLC cells. Bar diagrams represent the number of colonies of NSCLC cells treated with different doses of MH (**E**–**G**). Statistical analysis was performed using Student *t*-test with Graphpad Prism version 5 software. **, *p* < 0.01; ***, *p* < 0.001.

Cell viability assays were used to evaluate how effectively MH inhibited the proliferation of drug-sensitive (A549, A549-LUC, and H1299) and drug-resistant (A549-TR) cells. MH dose-dependently inhibited the proliferation of all tested lung cancer cells, with IC50 values ranging between 5 µM and 10 μM (Figure 2A–C; Supplementary Figure S1). MH demonstrated a similar IC50 (7.5 μM) against A549 and A549-LUC cells, while H1299 cells were more sensitive to MH (IC50, 5 μM). Interestingly, MH also exhibited significant dose-dependent antiproliferative activity against A549-TR cells, with an IC50 value of 10 μM.

#### 3.2.2. Effect on Colony-Forming Ability

The anticancer potential of a test substance can be determined by its ability to inhibit the colony formation of cancer cells. Treatment of A549, A549-TR, and H1299 cells with MH for 24 h dose-dependently inhibited colony formation. MH at 17.5 µM inhibited almost all colonies, whereas at a 10 µM concentration, MH significantly inhibited 48% and 24% of A549 (*p* < 0.001) and A549-TR (*p* < 0.01) colonies, respectively. On the other hand, H1299 colonies were significantly inhibited (35%; *p* < 0.001) by MH at 2.5 µM, with no colony formation observed at 10 µM (Figure 2D–G).

### 3.3. MH Exerts Cell Cycle Arrest by Inhibiting CDK4/6 and CDC2

**Figure 3:**
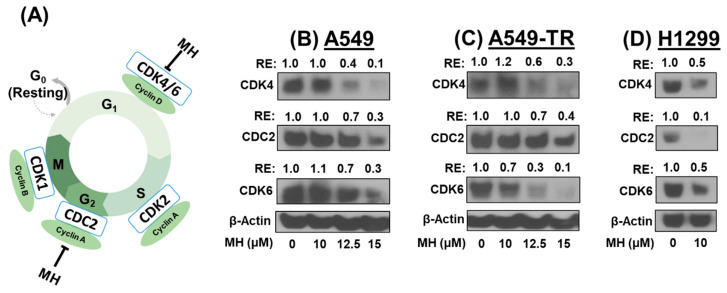
Effect of mahanine (MH) on cell cycle markers in lung cancer cells. (**A**) A schematic diagram illustrating MH’s effects on various phases of the cell cycle arresting the G_0_/G_1_ and G_2_ phases. Following 48 h MH treatment, protein lysates from NSCLC cells were analyzed by Western blot for cell cycle regulatory markers in A549 (**B**), A549-TR (**C**), and H1299 (**D**) cells. β-Actin was used as loading control. Numbers above the band depict relative expression (RE) compared to vehicle treatment.

MH has been reported to inhibit cancer cell growth by arresting the cell cycle at the G0/G1 and G2/M phases [14]. In this study, we assessed the impact of MH on cell cycle regulatory proteins, including CDK4/6 and CDC2 (Figure 3A). Treatment with MH led to the dose-dependent inhibition of CDK4/6 and CDC2 expression in both drug-sensitive (A549 and H1299) and drug-resistant (A549-TR) lung cancer cells (Figure 3). Compared to untreated cells, MH dose-dependently resulted in a significant (*p* < 0.01) reduction in CDK4/6 and CDC2 markers. MH inhibited cell cycle indicators, including CDK4/6 and CDC2, by 50–90% at 15 μM against A549 and A549-TR (Figure 3B,C) and 10 μM against H1299 cells (Figure 3D). These findings suggest that MH can halt lung cancer cell growth by directly inhibiting cyclin-dependent kinases.

### 3.4. MH Inhibit Anti-Apoptotic Markers

**Figure 4:**
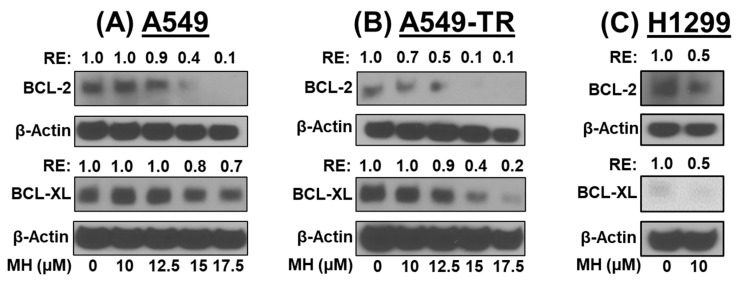
Effect of mahanine (MH) on apoptotic markers in A549 (**A**), A549-TR (**B**), and H1299 (**C**) cells. NSCLC cells were treated with MH for 48 h, and protein lysates were probed to assess the levels of apoptosis markers. β-Actin was used for equal loading. Numbers above each band depict relative expression (RE) compared to vehicle-treated cells. Figure 4 and Figure 5 share the same β-Actin blot for BCL-2 and Survivin in A549-TR cells.

Anti-apoptotic proteins Bcl-2 and Bcl-XL play important roles in preventing mitochondria-dependent cell death. Inhibition of these proteins significantly contributes to the activation of apoptosis in cancer cells [15]. The data (Figure 4) show that MH treatment dose-dependently inhibited both Bcl-2 and Bcl-XL in drug-sensitive (A549 and H1299) and drug-resistant (A549-TR) lung cancer cells (Figure 4A–C). MH at 17.5 μM exhibited 90% inhibition of BCL-2 (*p* < 0.001) and 30–80% inhibition of BCL-XL (*p* < 0.01 and *p* < 0.001) in A549 (Figure 4A) and A549-TR (Figure 4B) cells, respectively. In H1299 cells treated with 10 µM MH, both BCL-2 and BCL-XL were inhibited by 50% (*p* < 0.001) (Figure 4C).

### 3.5. MH Inhibits the MET/PI3K/AKT/mTOR Pathway

**Figure 5:**
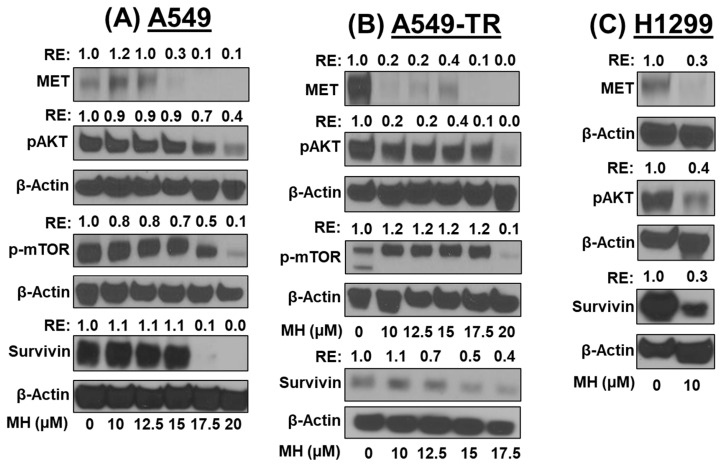
Effect of mahanine (MH) on the MET/PI3K/Akt/mTOR pathway. MH induces apoptosis in NSCLC cells by blocking the MET/AKT/mTOR/survivin signaling pathway. The impact of MH treatment on oncogene expression in NSCLC cells A549 (**A**), A549-TR (**B**), and H1299 (**C**) after 48 h is shown through immunoblotting analysis. Equal loading is represented by the levels of β-Actin. Numbers above the band depict the relative expression (RE) in comparison with vehicle-treated cells. Figure 4, Figure 5 and Figure 6 share the same β-Actin for a few protein markers.

The MET tyrosine kinase signaling pathway is highly active in various cancers, including lung cancer. MET stimulates the apoptosis-inhibitory protein survivin through the PI3K/AKT/mTOR pathway. Lung cancer cells, such as A549, A549-TR, and H1299, exhibit these oncogenic markers on their surface. Our findings reveal that MH treatment dose-dependently reduces the expression of MET, p-AKT, p-mTOR, and survivin in these cell lines (*p* < 0.001). Specifically, MH inhibits these markers by approximately 70% at 20 µM in A549 and A549-TR cells and 10 µM in H1299 cells (Figure 5A–C).

### 3.6. MH Inhibits the RAS/cMyc Pathway

**Figure 6:**
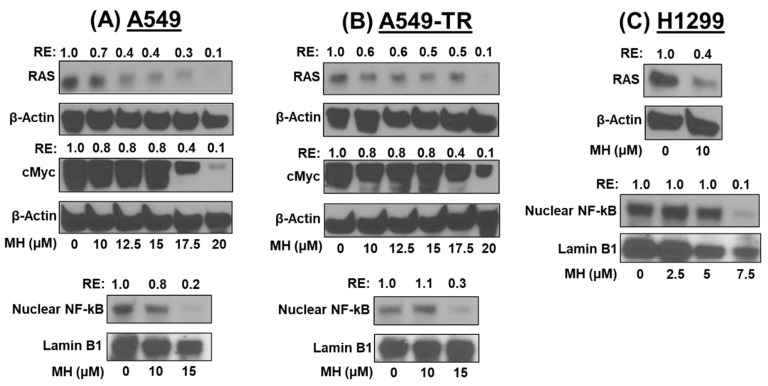
Effect of mahanine (MH) on RAS/cMYC and NF-kB. Cells were treated with MH for 48 h, and whole cell and nuclear lysates were prepared. The effect of MH on RAS/cMYC using whole cell lysates and NF-κB in nuclear extracts of NSCLC A549 (**A**), A549-TR (**B**), and H1299 (**C**) cells was determined by Western blot analysis. Equal loading was determined by the levels of β-Actin (whole cell lysates) and Lamin B1 (nuclear extracts). Numbers above each band depict the relative expression (RE) in comparison with vehicle-treated cells. Figure 5 and Figure 6 share the same β-Actin blots for p-mTor and RAS in both A549 and A549-TR cells.

Two of the most frequently activated oncogenes in carcinogenesis are cMyc and RAS, which collectively and individually regulate various cancer hallmarks, such as self-renewal, apoptosis, and proliferation. Studies indicate that inactivating both RAS and cMyc can halt the growth of lung cancer tumors. [16]. MH treatment dose-dependently downregulates RAS and cMyc, regardless of cell subtype (Figure 6A–C). At 17.5 μM, MH inhibits both RAS and cMyc by 90% in both A549 and A549-TR cells (*p* < 0.001), whereas 60% inhibition in RAS was observed in H1299 cells (*p* < 0.001) treated with 10 μM MH treatment.

### 3.7. MH Downregulates NF-κB

The oncogenic transcription factor NF-κB is a key mediator in the development of NSCLC. In this study, we investigated the effect of MH on nuclear NF-κB levels. The data (Figure 5C) show that MH treatment dose-dependently inhibited nuclear NF-κB levels in all the lung cancer cells tested. In A549 and A549-TR cells, MH at 15 μM decreased NF-κB levels by over 70%, while the same effect was evident in H1299 cells at a lower dose of 7.5 μM (Figure 6A–C).

### 3.8. MH Suppresses Orthotopic Lung Tumor Growth

**Figure 7:**
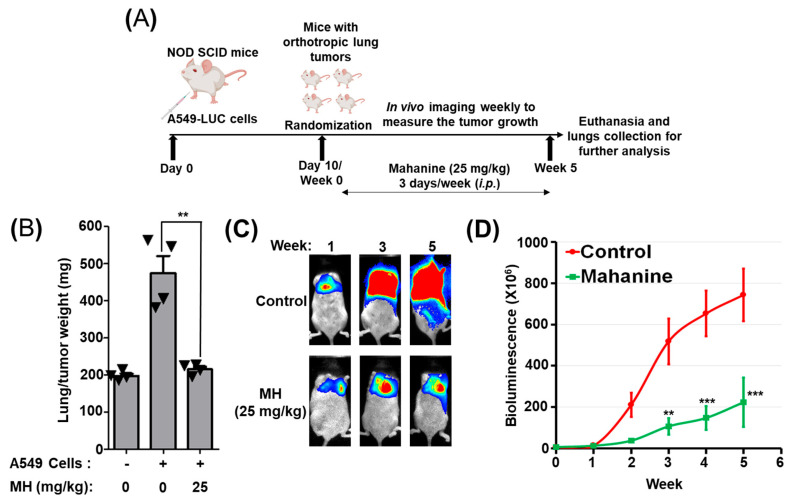
Effect of mahanine (MH) on the growth of orthotopic lung tumor growth. The line diagram depicts the experimental plan (**A**) and the effect of MH on tumorous lung weight (**B**), tumor progression in terms of luciferase signals (**C**), and growth inhibition (**D**) in female NOD Scid mice. Animals were inoculated with human Bioware Brite A549 Red-Luc cells (1.5 × 10^6^ cells) in 50 μL Matrigel via intrathoracic injection to develop orthotopic tumors. Ten days post-implantation, animals were imaged, randomly assigned to treatment groups, and administered intraperitoneal injections of either the vehicle or MH (25 mg/kg) three times per week. Tumor growth kinetics were monitored via bioluminescent imaging twice weekly. Prior to each imaging session, animals received an intraperitoneal injection of luciferin substrate (80 mg/kg) 15 min before imaging. Tumor burden was quantified in vivo using a Biospace Lab Imager. At the study’s endpoint, animals were euthanized, and the weights of excised tumor-bearing lungs were recorded. Data are expressed as mean ± SD, and statistical analysis was conducted using Student’s *t*-test; ** *p* < 0.01; *** *p* < 0.001.

The orthotopic lung tumor model is widely used to evaluate the anticancer activity of different therapeutic agents against lung cancer. To assess in vivo anti-tumor activity, NOD Scid mice were orthotopically inoculated with Bioware^®^ Brite A549 Red Fluc cells (1.5 × 10^6^). Based on live animal imaging of bioluminescent signals, the establishment of tumors in mouse lungs was confirmed over a 10-day period. Subsequently, over a five-week duration, untreated animals exhibited almost exponential lung tumor growth. MH treatment (25 mg/kg; 3 doses a week) significantly inhibited tumor growth (*p* ≤ 0.001) (Figure 6). The mean tumor volume, as represented by the bioluminescent signals in MH-treated mice, was 222 × 10^6^ photons compared to 743 × 10^6^ in the control group, which corresponds to an average reduction of about 70% in tumor volume (*p* < 0.001) (Figure 7C,D). Lung weight, reflecting tumor burden, was also significantly reduced (*p* < 0.01) with MH treatment (214 ± 14 mg) compared with the control (473 ± 93 mg) (Figure 7B).

## 4. Discussion

In this study, we demonstrate the efficacy of MH in inhibiting the proliferation of various subtypes of drug-sensitive and drug-resistant NSCLC cells. Phytocompounds have been shown to target distinct molecular pathways through polypharmacology, thereby enhancing their therapeutic effectiveness [17]. Our study suggests that MH employs a polypharmacology strategy against lung cancer. We observed a dose-dependent downregulation of MET and its downstream signaling pathway markers, including p-AKT, p-mTOR, and survivin, in all NSCLC cell lines. This downregulation led to the inhibition of cell proliferation and colony formation. These findings demonstrate MH’s potential as a promising therapeutic agent for targeting multiple pathways involved in NSCLC progression. Our results align with those of Chatterjee et al., 2015 [18], who showed that MH inhibited lung cancer cell progression by downregulating p-mTOR and p-AKT in vitro.

Survivin, the smallest member of the inhibitors of the apoptosis protein family, plays a critical role in regulating cell division and apoptosis binding to X-linked inhibitors of apoptosis proteins [19,20]. Cells can avoid apoptosis and survive by activating survivin through several mechanisms. In this study, MH significantly modulated the levels of survivin. Although several other chemopreventive agents have altered survivin expression [21], ours is the first report highlighting the effect of MH on survivin in lung cancer.

MET is a transmembrane protein that mediates the tyrosine kinase receptor activated by hepatocyte growth factor (HGF) [22]. Physiologically, MET plays a critical role in tissue remodeling and morphogenesis, and its dysregulation mediates migration, apoptosis, and proliferation, which are associated with several cancers [23]. Upon HGF binding, MET receptors dimerize, leading to the phosphorylation of tyrosine residues and subsequent phosphorylation of intracellular docking sites. This initiates downstream signaling pathways, including the phosphoinositide 3-kinase (PI3K)/AKT and NF-κB pathways.

The PI3K/AKT/mTOR signaling pathway is frequently activated in NSCLC and plays a crucial role in tumor development by promoting cell survival, growth, proliferation, and migration [24]. Upon the binding of ligands to tyrosine kinase receptors, such as MET, PI3K, EGFR, or insulin-like growth factor 1 receptor, PI3K is activated, which catalyzes the conversion of phosphatidylinositol bisphosphate into phosphatidylinositol triphosphate [25]. This conversion drives the recruitment of AKT to the plasma membrane, where it phosphorylates multiple downstream targets involved in regulating cell survival, proliferation, motility, and metabolism. mTOR, a serine/threonine kinase, functions within two distinct complexes, mTORC1 and mTORC2, each with unique structural and functional roles [26].

AKT activation stimulates mTORC1, promoting cell growth by altering cellular energy status [27]. Our current study suggests that MH can potentially prevent the growth of lung cancer by downregulating MET and its downstream signaling markers, including p-mTOR, p-AKT, and NF-κB. Further studies are needed to ascertain the role of MH in regulating the MET-mediated inhibition of NF-κB.

In our study, we found that MH inhibited the growth of lung cancer cells by lowering the expression of cMYC and cell cycle regulatory proteins CDK4/6 and CDC2. In conjunction with D-type cyclins, CDK4 and CDK6 phosphorylate the tumor suppressor protein Rb, accelerating the cell cycle through the G1 restriction point [28]. CDK4/6 are considered key targets for cancer drug development [29]. Reports suggest that inhibiting cMyc halts lung cancer cell growth by inhibiting cyclin-dependent kinases, such as CDK4/6 and CDC2 [30]. RAS mutations are common in cancer tumorigenesis. It is noteworthy that A549 lung cancer cells harbor the KRAS G12S mutation, while H1299 cells lack KRAS mutations. Nevertheless, our study finds that MH suppressed both KRAS-mutated (A549) and KRAS wild-type (H1299) cells, indicating that the effect of MH on lung cancer is independent of KRAS inhibition.

Anti-apoptotic proteins, like Bcl-2 and Bcl-XL, are often overexpressed in cancer, leading to an anti-apoptotic phenotype that allows cancer cells to evade cell death [31]. Bcl2 and Bcl-XL inhibit the loss of mitochondrial membrane potential mediated by Granzyme B, Bcl-XL, and BNIP3, as well as caspase-mediated apoptosis, thereby promoting cancer cell survival. Our findings provide additional evidence that MH induces apoptotic cell death by inhibiting Bcl2 and Bcl-XL in drug-sensitive and drug-resistant lung cancer cells in a dose-dependent manner.

Activation of NF-κB has been associated with chronic inflammation and cancer development, contributing to the onset and progression of various human malignancies. NF-κB has been reported to be associated with various hallmarks of cancer, including proliferation (Myc), survival (Survivin), and angiogenesis (vascular endothelial growth factor or VEGF) [32]. In many cancer cells, NF-κB remains inactive in the cytoplasm through its interaction with IκB protein. Various stimuli at the tumor site, including stress, growth factors, and cytokines, activate NF-κB. Once activated, NF-κB translocates to the nucleus, where it binds to DNA and regulates the expression of several genes responsible for cancer progression [33]. NF-κB activates anti-apoptotic proteins, such as the cellular inhibitor of apoptosis proteins 1 and 2, X-linked inhibitor of apoptosis protein, and Bcl2 family proteins [34].

We investigated the effect of MH on NF-κB expression. Our results show that MH dose-dependently inhibits NF-κB expression in the cell nucleus. The accumulation of pro-inflammatory cytokines at the tumor site enhances NF-κB activation, creating a pro-tumorigenic microenvironment. Additionally, NF-κB promotes tumor metastasis by inducing epithelial–mesenchymal transition (EMT) through the activation of transcription factors, such as Twist1 and Snail, and by regulating selectins and integrins involved in cancer cell migration and colonization at distant sites. It also regulates tumor angiogenesis by inducing the expression of fibroblast growth factor, interleukin 8 (IL-8), and matrix metalloproteinase-9 (MMP-9) [35,36]. A reduction in NF-κB expression may contribute to the apoptosis activation and inhibition of lung cancer metastasis and angiogenesis.

The inoculation of cancer cells into the lung cavity to produce orthotropic tumors is a commonly used pre-clinical model to test the antitumor activity of agents within the tumor microenvironment [37]. In this study, we found that MH (25 mg/kg) effectively (≈70%) suppressed the growth of orthotropic lung tumors, which can be attributed to its impact on multiple oncogene targets, induction of apoptosis, and cell cycle arrest.

In summary, our study suggests that MH holds promise as a novel drug candidate for the treatment of lung cancer. Encapsulation of MH into nanoparticles, such as biocompatible exosomes [38,39], could potentially enhance its efficacy through targeted delivery to the tumor site.

## Data Availability

Data will be available on demand.

## Supplementary Materials

The following supporting information can be downloaded at: https://www.mdpi.com/article/10.3390/cancers16213572/s1, Figure S1: Effect of mahanine (MH) on lung cancer cell proliferation. Cell viability of A549-LUC cells was determined by luciferase assay after treatment with MH for 72 h. Data represents mean ± S.D. of 3 independent experiments.

## Abbreviations

A549-TR: Taxol-resistant A549 lung cancer cells; BCL-2, B-cell leukemia/lymphoma 2; BCL-XL, B-cell lymphoma-extra large; CDC2, Cell division control 2; CDK4, Cyclin-dependent kinase 4; CDK6, Cyclin-dependent kinase 6; cMyc, Myelocytomatosis oncogene; EGFR, Epidermal growth factor receptor; FBS, Fetal bovine serum; HGF, Hepatocyte growth factor; LC-MS, Liquid chromatography–mass spectrometry; MET, Mesenchymal–epithelial transition factor receptor; MH, Mahanine; MTT, 3-(4,5-Dimethylthiazol-2-yl)-2,5-diphenyltetrazolium bromide; NF-κB, Nuclear factor kappa-light-chain-enhancer of activated B cells; NRM, Nuclear magnetic resonance; NSCLC, Non-small cell lung cancer; p-mTOR, Phosphorylated mammalian target of rapamycin; p-AKT, Phosphorylated protein kinase B; RAS, Rat sarcoma viral oncogene; KRAS, Kirsten rat sarcoma viral oncogene homolog; RT, Retention time; TLC, Thin-layer chromatography; UPLC, Ultra-performance liquid chromatography.
