# Supplementary material for: Oncogene Downregulation by Mahanine Suppresses Drug-Sensitive and Drug-Resistant Lung Cancer and Inhibits Orthotopic Tumor Progression
Source: Cancers (Basel). 2024 Oct 23;16(21):3572. doi: 10.3390/cancers16213572 (PMC11545155; doi:10.3390/cancers16213572)
Supplement: Supplementary file 1 [file cancers-16-03572-s001.zip › cancers-3251161-supplementary.pdf]

Supplementary figure

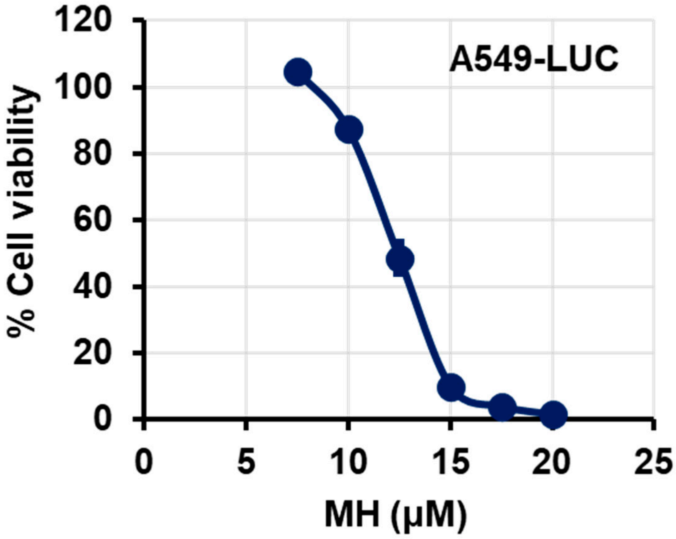

Figure S1. Effect of mahanine (MH) on lung cancer cell proliferation. Cell viability of A549-LUC cells was determined by luciferase assay after treatment with MH for 72 h. Data represents mean ± S.D. of 3 independent experiments.
